# Children’s questions and teachers’ responses about COVID-19 in Türkiye and the US

**DOI:** 10.1371/journal.pone.0307475

**Published:** 2024-07-22

**Authors:** Burcu Ünlütabak, Graciela Trujillo Hernandez, İlayda Velioğlu, David Menendez, Karl S. Rosengren

**Affiliations:** 1 Department of Psychology, Yeditepe University, Istanbul, Türkiye; 2 Department of Psychology, University of Rochester, Rochester, NY, United States of America; 3 Department of Psychology, Kadir Has University, Istanbul, Türkiye; 4 Department of Psychology, University of California-Santa Cruz, Santa Cruz, California, United States of America; 5 Department of Brain and Cognitive Sciences, University of Rochester, Rochester, NY, United States of America; RMIT University, VIET NAM

## Abstract

Question-asking is a crucial tool for acquiring information about unseen entities, such as viruses; thus, examining children’s questions within the context of COVID-19 is particularly important for understanding children’s learning about the coronavirus. The study examined 3-12-year-old children’s questions and teachers’ responses about the COVID-19 pandemic in Türkiye, a non-Western developing context, and the United States, a Western cultural context. A total of 119 teachers from Türkiye and 95 teachers from the US participated in the study. Teachers completed an online survey consisting of a demographic form and a questionnaire asking them to report three questions about COVID-19 asked by children in their classrooms and their responses to these questions. We analyzed children’s questions and teachers’ responses for their type and content and examined demographic factors associated with children’s questions and teachers’ responses. Consistent with the literature, children from Türkiye asked fewer explanation-seeking (i.e., why/how) questions than children from the United States. Children asked questions about viruses and precautions. Teachers responded to children’s questions realistically in both countries. The findings have important implications for how children gain knowledge from teachers when discussing health, disease, and virus topics in two countries.

## Introduction

The COVID-19 pandemic caused considerable confusion and uncertainty in the world, causing adults and children to ask many questions about the virus and safety precautions put in place to diminish the risk of infection. It was difficult for children to make sense of these changes, and they had to rely on information from trustworthy informants around them (i.e., parents and teachers). Although there has been considerable focus on parents/caregivers as informants, few studies examined the role of teachers as informants on topics that are peripheral to the main classroom content. However, children often spend considerable time interacting with teachers. In this study, we extend previous research on children’s questions with parents [1–3] and examine children’s questions about COVID-19 directed toward their teachers and how the teachers responded to these questions in Türkiye and the United States (US).

### Children’s question-asking behavior

Studies show that children actively seek information within everyday parent-child conversations and insist on getting answers [4–6]. Children’s question-asking provides insight into children’s level of conceptual knowledge at different ages [7]. Toddlers’ questions mainly target factual information, while preschoolers start asking for causes [5, 8, 9]. Parents respond to children’s questions and provide explanatory responses [4, 5].

### Children’s questions to teachers

Recent research highlights the importance of teacher-child dialogue in classroom contexts. Studies show that children engage in question-answer exchanges about scientific topics [10–13]. However, studies on teacher-child interactions in the classroom report somewhat mixed findings about teachers’ responses to children’s questions.

An observational study in a US classroom found that teachers provided explanatory answers to children’s information-seeking questions [14]. Likewise, a survey study in the US showed that the more experienced teachers were more likely to report that they would provide exploratory opportunities to children [11]. On the other hand, in a qualitative interview study with Turkish teachers, findings showed that teachers had difficulty providing explanatory responses to children’s challenging questions about scientific topics (i.e., nature, religion, reproduction) in the classroom. Only one-fourth of the teacher’s responses included realistic answers to such questions [13].

These findings indicate that teachers, like parents, frequently interact with children and are influential informants. Teachers also have a more explicit pedagogical role than parents, potentially leading children to ask different types of questions. In a study conducted in the US, most parents acknowledge the importance of teachers in furthering their child’s knowledge (even about topics like nutrition that might not be directly taught in school [15]. Therefore, examining children’s questions and teachers’ responses about COVID-19 may show us how children seek information from teachers and how teachers guide children’s learning.

### Children’s questions about the COVID-19 pandemic

Research conducted in Türkiye and the US on children’s questions and parents’ responses about the COVID-19 pandemic indicates that since the beginning of the pandemic, parents have observed changes in the content of child-initiated questions and parents’ responses. These findings in these two sociocultural contexts suggest that the COVID-19 pandemic led children to ask questions to their parents about the virus, lifestyle changes, school, and safety, and parents provided informative answers. These studies also highlighted some context-specific differences with regard to the content and style of children’s question-asking behavior during the pandemic [1–3].

Virus-related conversations revolve around hidden/unobservable concepts, and our knowledge of the virus changed throughout the pandemic. These issues made it challenging for parents to answer children’s questions. However, parents in Türkiye and the US who felt they had enough knowledge were more likely to report explanations [1, 3]. Although these studies provide insights into children’s learning about the pandemic, they neglect teachers’ role as influential informants in children’s lives. While there have been studies investigating children’s questions to teachers about science, we know of no studies examining children’s questions and teacher’s responses about the COVID-19 pandemic. We addressed this gap by surveying teachers of 3-12-year-olds about children’s COVID-19-related questions and their responses in Türkiye and the US.

### Cultural differences in questions

Research in developmental psychology has predominantly included samples from the US, other English-speaking countries, or Western European countries [16], limiting our understanding of the generalizability of past results. Question-asking behavior is a social process requiring interactions with adults in culturally appropriate ways [17, 18]. Western and non-Western countries have different culture-specific values and practices. Specifically, values of independence and autonomy are more commonly emphasized in Western countries, whereas respect for elders and deference are more commonly highlighted in non-Western countries [19]. These values may be associated with differences in children’s question-asking behavior, with more explanation-seeking questions occurring in Western countries vs. fewer explanation-seeking questions in non-Western countries [20].

In a cross-cultural study, Gauvain, Munroe, and Bebee examined children growing up in non-industrialized societies (Garifuna in Belize, Logoli in Kenya, Newars in Nepal, and Samoans in America Samoa) and found that they asked fewer explanation-seeking questions (5% of all questions) than children in industrialized countries (the US and the UK) (26–30% of all questions) [5, 6, 21]. Likewise, a study conducted with children in Türkiye showed that 3-to-6-year-old children asked fewer explanation-seeking questions (22% middle-SES and 10% low-SES) than children in Western countries [22]. These findings highlight the need for more cross-cultural research examining children’s questions.

Türkiye and the US are similar in that they both have great cultural and ethnic diversity with minority and immigrant populations. However, the US emphasizes individualistic values and promotes independence and self-reliance as socialization goals [19]. Türkiye is going through rapid urbanization and industrialization and exhibits a blend of Western and non-Western values across traditional and modern sectors of society [23, 24]. Thus, we can observe differences in how children view question-asking (Is it appropriate to ask elders? What types of questions are effective for eliciting information?) and how teachers respond to them.

### Education systems in the context of COVID-19

#### Türkiye

Türkiye has a centralized education system with 12 years of compulsory education for children between 6 and 18. The government supports compulsory education, freely available in public schools [25]. Preschool education is not part of compulsory education; it is optional. Parents can send their children to private preschools. Public schools have optional kindergarten classes starting at age 5. Recently, preschool enrollments have been increasing, and more importance is given to early childhood education due to urbanization, economic development, and women empowerment [25].

Teachers are expected to have a minimum of a bachelor’s degree [26]. Teachers are recruited for public schools via a centralized exam for public personnel selection. Teachers are assigned to different public schools depending on their scores and the branch (e.g., math, science, reading). Schools in the villages are prioritized in the assignment. Teachers work on probation for a year and then can take a centralized exam to gain permanent status [25].

After the declaration of the worldwide pandemic, the Turkish Ministry of Education announced the full closure of schools, with transition preparations for online education. A week later, online education started in Türkiye via the Education Information Network (EBA) platform. EBA has been used for synchronous education and asynchronous activities for children. A national EBA TV broadcasts at all levels of classes on public channels, making educational material accessible to all children from diverse backgrounds [27]. In-person education restarted in the classrooms in the Fall of 2021.

#### The US

In the US, education is compulsory for all children. However, there is no centralized system like in Türkiye. Rather, each state determines the age of required school attendance, which varies by state in both the starting and ending ages. Specifically, the age children must start attending school varies from 5 to 8 years, and the age at which children may discontinue schooling varies from 16 to 18. Generally, free public education is available from kindergarten (Ages 4 to 5) to 12th grade (ages 17 to 18). The content and format of schooling are determined by a mixture of local, state, and federal regulations [28]. Most kindergarten through twelfth-grade children attend public schools (~87%) funded by local taxes. Approximately ten percent of children in these grades attend private schools primarily affiliated with a particular religion. Roughly 3% of children in the US are schooled at home [29].

Teacher training in the US follows a similar pattern of limited central regulations, with most teaching requirements determined at the State and local levels. Most states require teachers to have a bachelor’s degree in education or the specific field they intend to teach (e.g., mathematics, science, English). In some states, a college-level teaching certificate is all that is required to be a classroom teacher in a public school. Public school teachers must obtain classroom experience before being hired for a full-time teaching position [30]. In private schools, the individual schools determine the requirements for teaching, and teachers often are not required to have a teaching license, only knowledge of a particular subject matter [31].

Similar to schools in Türkiye, school districts in the US (over 70% of public and private schools) switched to remote learning from 2020 until 2021. During this period, technology and internet assistance were provided to the families. Students had the opportunity to attend summer schools and work with private tutors in the Summer of 2021 [32]. The decision for when to return to in-person classes was also determined locally, with most schools returning to in-person classes for at least part of the week in Fall 2021.

### Research aims

1. Identifying the form and content of children’s questions about the COVID-19 pandemic addressed to their teachers in Türkiye and the US.
2. Examining the quality of teachers’ responses that teachers provided for children’s questions in Türkiye and the US.
3. Understanding the influence of demographic factors (e.g., age, gender, grade level) on children’s questions about COVID-19 and teachers’ responses.
4. Exploring the relationship between children’s COVID-19-related questions and teachers’ responses in Türkiye and the US.

### Specific research questions and hypotheses

1. What is the form and content of 3- to 12-year-old children’s questions about COVID-19 in Türkiye and the US? Which demographic factors are associated with children’s questions? We expected that children in higher grades would ask more school and virus questions while children in lower grades would ask about lifestyle changes. Children in the US would be more likely to ask explanation-seeking questions than children in Türkiye [3, 22]. In both contexts, virus and precaution questions would be the most common content [1].
2. What is the form and content of teachers’ responses to children’s questions in Türkiye and the US? Which demographic factors are associated with teachers’ responses? We expected teachers to provide realistic responses to children. We expected that teachers in Türkiye would be more likely to refer to rules and authority than teachers in the US because of the collectivistic emphasis on interdependence and respect for elders in Türkiye [19, 20]). Teachers who felt more knowledgeable would be more likely to provide realistic responses [1, 3].
3. What is the relationship between children’s questions and teachers’ responses? We expected that teachers would be more likely to provide appropriate explanations to children’s why/how questions than other types of questions [4].

## Materials and methods

### Participants

#### Turkish sample

The approval was received from the Institutional Review Board (IRB) at Nuh Naci Yazgan University (Institutional Review Board of Nuh Naci Yazgan University, Approval No: 2024/5458, Date: 13.12.2021). One hundred thirty teachers over the age of 18 from Türkiye were recruited. Unlike the US, there is no crowdsourcing website like Amazon Mechanical Turk actively providing survey participation for researchers in Türkiye. Thus, teachers were recruited via social media, online forums, and communication applications and via directly reaching out to teachers via schools. The Turkish survey was prepared by using Google Forms. The questionnaires were filled out online by all teachers and they were informed about all aspects of the study, and informed consent was obtained from all participants before the online survey began. The data collection process, which started on December 21, 2021, took approximately 1.5 months and ended on February 1, 2022. Eleven participants were excluded either because they did not respond appropriately to the screening questions (e.g., “I live in Türkiye,” “I work as a teacher,” and “I teach children under the age of 13”) or due to missing data. In the final sample, there were one hundred nineteen participants. Most of the teachers taught at schools in urban areas (n = 80, 67.2%). The remaining teachers taught at schools in subprovinces (n = 20, 16.8%) or villages (n = 16, 13.4%). See Table 1 for demographic characteristics.

**Table 1:** Participants’ demographic characteristics.

	Sample in Türkiye		Sample in the US	
	n	%	n	%
Gender				
Woman	93	79.5	50	52.6
Man	24	20.5	44	46.3
Non-Binary	-	-	1	1
Age				
21–30	40	34.8	45	47.4
31–40	36	31.3	32	33.7
41–50	30	26	14	14.7
51–61	9	7.8	4	4.2
Class size				
1–15	30	25.9	14	15.9
16–30	76	65.5	64	72.7
31–50	10	8.6	9	10.2
50–70	-	-	1	1.2
Education level				
High School			3	3.4
2-year degree	1	0.8	2	2.2
Bachelor’s	108	92.3	48	53.9
Master’s	8	6.8	33	37.1
PhD			3	3.4
Years of experience				
0–10	51	44.3	72	75.8
11–20	33	28.7	16	16.8
21–30	28	24.3	6	6.3
31–42	3	2.6	0	0
School Type				
Public	89	76.7	55	57.9
Private	23	19.8	29	30.5
Home	-	-	6	6.3
Charter	-	-	3	3.2
Other	4	3.4	2	2.1
Grade level taught				
Preschool	25	21	4	4.2
1st - 4th grade (Primary)	49	41.2	63	66.3
5th - 8th grade (Secondary)	41	34.4	27	28.4

#### US sample

IRB approval was received from the University of Rochester (Institutional Review Board of University of Rochester, Study ID: STUDY00006672, Date: 11.19.2021). One hundred fifty-six teachers over the age of 18, living in the US, who taught children younger than 13 years of age were recruited using Amazon’s Mechanical Turk, an academic research tool for survey administration. Participants were informed about all aspects of the study and completed informed consent forms prior to the online survey. The English survey was prepared using Qualtrics. The data collection process started on January 25th and ended on February 7th, 2022. Participants were paid $2 for completing the survey. Twice in the survey, teachers were asked to report the grades that they taught. Once as an open-ended question and once as a close-ended question. Anyone who did not report that they taught classrooms between preschool to 8th grade was excluded from analyses (n = 61). The final sample resulted in 95 teachers from 31 of the 50 states. Most of the sample reported teaching in a school located in an urban community (*n* = 46, 60%), with smaller percentages of teachers teaching in schools in suburban (*n* = 20, 26%) and rural (*n* = 11, 14%) communities.

Grade levels corresponding to primary, secondary education are different in Türkiye and the US. Türkiye implements a 4(primary) + 4(lower secondary) + 4 (higher secondary) education system in which each level lasts 4 years. In the US, primary education is 4 years, lower secondary education is 3 years and higher secondary education is 4 years. For the ease of presentation, we provided frequencies and percentages using 4-year-segments on Table 1.

### Materials

The online survey consisted of 32 questions about children’s questions, teachers’ responses, and demographic information.

#### Children’s questions and teacher’s responses

Teachers were asked to report three questions children had asked about COVID-19 since the beginning of in-person education and how they responded to these questions.

#### Demographics questionnaire

Teachers were asked to report their age, gender, education level, years of experience and biology knowledge, the type of school teachers currently work at (e.g., public or private), the location of the school (e.g., village, town, or city), and the class size. Questions about the COVID-19 policies of the schools and any mask and vaccination requirements were asked. Finally, teachers were asked whether they had obtained any personal training or whether the school provided any educational training about the COVID-19 pandemic.

### Qualitative coding

Children’s questions and teachers’ responses were coded using Menendez et al’s coding categories [1]. Children’s questions were coded for linguistic type: 1) What, 2) Why/How, 3) Yes/No, 4) Tag, 5) Permission request, and 6) Other. Children’s questions were coded for the content: 1) Lifestyle Changes, 2) Preventive Measures, 3) Safety, 4) School, 5) Symptoms, 6) Time, 7) Vaccination, 8) Virus, 9) Other/No Question.

Teachers’ responses were coded for content: 1) Authority, 2) Realistic, 3) Reassurance, 4) Religious, 5) Other, and 6) No Elaboration. Explanations were coded for causal content: 1) mechanism, 2) prior cause, 3) consequence, 4) conditional, and 5) Non-explanatory/Precondition [4].

Two research assistants from Türkiye and two from the US coded the data in their respective countries. For inter-coder agreement, the two coders double-coded 100% of the data. Disagreements were resolved via discussion. Due to low to moderate interrater reliability in several content categories, a third coder also coded the data and resolved the disagreements. In addition, Cohen’s kappa values for the Yes/No category are lower than 100% agreement due to initial discrepancies in coding permission-asking questions as Yes/No questions by one coder. These discrepancies were resolved via discussions. All Cohen’s Kappa (κ) values, examples, and code descriptions are presented in Tables 2–4.

**Table 2:** Codes for children’s question types with the kappa and prevalence in Türkiye and the US.

			Data in Türkiye		Data in the US	
Category	Definition	Examples	Kappa	n (%)	Kappa	n (%)
What	Wh- Questions such as what, where, who.	*“What is coronavirus*?*”*	0.96	103 (29.4%)	0.92	61 (22%)
Why/How	Questions requesting causal information such as why, how, and what would happen if.	*“How are we wearing a mask*?*”*	0.95/0.97	65 (18.6%)	0.98/0.98	88 (31.8%)
Yes/no	Questions asking for a yes/no answer	*“Are you vaccinated*?*”*	0.87	153 (43.7%)	0.91	93 (33.6%)
Tag	Questions with a statement seeking confirmation	*“The pandemic is going to end*, *isn’t it*?*”*	0.8	8 (2.3%)	1	0 (0%)
Permission	Questions asking for permission	*“Can I go to the restroom*?*”*	0.7	15 (4.3%)	0.61	5 (1.8%)
Other	Sentences reported by teachers but do not include any question.	* *	NA	-	0.94	30 (10.8%)

**Table 3:** Codes for the content of children’s questions with the kappa and prevalence in both Türkiye and the United States.

			Data in Türkiye		Data in the US	
Category	Definition	Examples	Kappa	n (%)	Kappa	n (%)
Lifestyle Changes	Questions involving lifestyle changes with the pandemic	*Can we go to the cinema*, *to the theater*, *to travel*?	0.81	22 (6%)	0.8	12 (4.3%)
Preventive Measures	Questions about the protective measures taken due to the pandemic	*Why do we cover our mouth when coughing*?	0.82	93 (25.5%)	0.94	70 (25.2%)
Safety	Questions about the safety of oneself, family, and loved ones	*Do we die when we get Covid-19*?	0.53	29 (8%)	0.75	37 (13.3%)
School	Questions about the changes in school due to pandemic	*Will schools close again*?	0.97	40 (11%)	0.77	19 (6.9%)
Time	Questions about how long the pandemic will last or when it will end	*When will it be over*?	0.91	63 (17.3%)	0.94	38 (13.7%)
Vaccination	Questions about mechanism, safety and necessity of the vaccine	*Will we get vaccinated too*?	0.98	28 (7.7%)	0.95	16 (5.8%)
Virus	Questions about the viruses, symptoms, and transmission	*Does it also infect children*?	0.79	82 (22.5%)	0.8	62 (22.4%)
Other	Statements or questions that does not fit into any category.	*How possible*?	0.67	7 (1.9%)	0.77	8 (2.9%)

**Table 4:** Content codes for teachers’ responses and explanations with the kappa and prevalence in Türkiye and the US.

			Data in Türkiye		Data in the US	
Content	Definition	Examples	Kappa	n (%)	Kappa	n (%)
Mutually Exclusive						
Authority^4^	Answers with official institutions, government, school administration	*You should listen to the advice of doctors*.	0.54	9 (2.5%)	0.8	13 (4.8%)
Realistic	Answers that are based on scientifically correct information	*You need to wear a mask because this is the best way to protect yourself from a disease*.	0.67	232 (65.2%)	0.8	112 (41.2%)
Supernatural	Answers that refer to non-scientific, unexplainable concepts	*Is COVID-19 a monster*?	-	0 (0%)	1	1 (0.4%)
Other	Answers that we could not classify under any category	*Because of their ignorance*.	0.81	16 (4.5%)	0.9	9 (3.2%)
No elaboration	Responses with no explanations, usually with short yes/no answers	*Yes *	0.75	79 (22.2%)	0.83	138 (50.7%)
Non-mutually Exclusive						
Reassurance	Answers that provide comfort and support to children	*You do not have to be afraid*.	0.63	42 (11.8%)	0.84	26 (9.6%)
Religious	Answers referring to religion	*By the grace of God*, *nothing (bad) will happen*.	-	5 (1.4%)	1	1 (0.4%)
Causal Content						
Mechanism	Descriptions of the steps necessary for an event to occur.	*It passes by touching*, *kissing*, *hugging*, *and using common items*.	0.8	26 (14.5%)	0.65	24 (18%)
Prior Cause	Answers including prior reasons for an event to happen	*We get vaccinated to be protected from Covid-19 and to get over it more easily*.	0.79	93 (51.9%)	0.79	18 (13/5%)
Consequence	Answers including consequences after an event has occurred	*The mask prevents you from infecting other people*.	0.49	34 (19%)	0.8	80 (60.1%)
Conditional	Answers that condition the occurrence of an event.	*If you pay attention to your distance and follow the rules*, *the risk will decrease*.	0.76	44 (24.6%)	0.84	42 (31.6%)
Non-explanatory/Precondition	Answers that emphasize prerequisites.	*If we are careful*, *we will not be (sick)*.	-	4(2.2%)	0.8	2 (1.5%)

### Data analysis plan

**G**eneralized linear mixed-effects models (GLMM) were conducted using the R Studio Statistical Analysis Program. Base R functions and the plyr package [33] were used for data preparation. The plyr package allowed us to structure our data frames. The lme4 [34], the car [35], the lmSupport packages [36] for data analyses were used for conducting generalized linear models and interpreting the results.

In the models for children’s questions, 1) child grade and 2) class size, 3) teacher’s age, 4) teacher’s gender, and 5) school type (public or private) were entered as predictors. In the models for teachers’ responses, 1) child grade, 2) class size, 3) teacher’s age, 4) teacher’s gender, 5) school type (public or private), 6) teacher’s education level, 7) teacher enough knowledge, 8) teacher biology knowledge, 9) COVID-19 school training, 10) COVID-19 personal training were entered as predictors. We decided to include teachers’ age but not teachers’ years of experience in the models because they were highly correlated. (Türkiye r = .97; US r = .70). Teachers’ age appeared to be a more critical predictor as it encompasses teachers’ experience and appearance as informants.

## Results

Whether the types and content of children’s questions and teachers’ responses varied by demographic factors was examined. Then, the relationship between children’s questions and teachers’ responses was explored. For each analysis, the data from the teachers in Türkiye were presented first, followed by the data from teachers in the US. At the end of each section, a summary of findings highlighting the similarities and differences between the two countries was included.

### Children’s questions

Teachers in Türkiye reported 350 questions, and teachers in the US reported 277 questions asked by their children about the COVID-19 pandemic.

#### Teachers in Türkiye

Teachers in Türkiye reported that most of the children asked yes/no questions (*n* = 153, 43.7%), followed by what (*n* = 103, 29.4%), how and why (*n* = 65, 18.6%), permission (*n* = 15, 4.3%) and tag (*n* = 8, 2.3%) questions. Only 18.6% of children’s questions were explanation-seeking (i.e., why-how, *n* = 65).

Children most frequently asked about preventive measures (*n* = 93, 26.6%) and the virus (*n* = 82, 23.4%), followed by time (*n* = 63, 18%), school (*n* = 40, 11.4%), vaccination (*n* = 28, 8%), safety (*n* = 29, 8.3%) and lifestyle changes (*n* = 14, 4%). Six teachers reported that children did not ask any questions about COVID-19.

In lower grades, children were more likely to ask explanation-seeking questions than in higher grades (*OR* = .38, *χ2* (1, *N* = 108) = 10.18, *p* = .001). In contrast, children were more likely to ask yes/no questions in higher grades than in lower grades (*OR* = 1.70, χ2 (1, *N* = 108) = 9.17, *p* < .01). Moreover, teachers in private schools were more likely to report that children asked explanation-seeking questions than teachers in public schools (*OR* = 3.07, *χ2* (1, *N* = 108) = 3.96, *p* < .05). Teachers in smaller classes were less likely to report that children asked explanation-seeking questions than teachers in larger classes (*OR* = 1.06, *χ2* (1, *N* = 108) = 4.54, *p* < .05). Teacher’s age and gender were not significant. For content, in higher grades, teachers were more likely to report questions about time (*OR* = 1.59, *χ2* (1, *N* = 110) = 4.98, *p* < .05), school (*OR* = 4.01, *χ2* (1, *N* = 110) = 9.40, *p* < .01) and vaccination (*OR* = 3.05, *χ2* (1, *N* = 110) = 7.51, *p* < .01).

#### Teachers in the US

Teachers in the US reported that children mostly asked yes/no questions (*n* = 93, 33.6%), followed by how/why questions (*n* = 88, 31.8%), what questions (*n* = 61, 22%), and permission questions (*n* = 5, 1.8%). Of all questions, 31.8% were explanation-seeking. Similar to the Turkish sample, children in the US mostly asked about preventive measures (*n* = 70, 25.3%), followed by virus (*n* = 62, 22.4%), time (*n* = 38, 13.7%), safety (*n* = 37, 13.4%), school (*n* = 19, 6.9%), vaccination (*n* = 16, 5.8%), interpersonal relations (*n* = 8, 2.9%) and lifestyle changes (*n* = 4, 1.4%).

Older teachers were more likely to report that children asked permission (*OR* = 1.15, *χ2*(1, *N* = 85) = 8.37, *p* < .01), yes/no questions (*OR* = 1.94, *χ2*(1, *N* = 85) = 4.04, *p* < .05), and less likely to ask what questions (*OR* = .94, *χ2*(1, N = 85) = 6.93, *p* < .01). Women teachers were more likely to report that children asked yes/no questions (*OR* = 1.94, *χ2* (1, *N* = 85) = 4.04, *p* < .05). Other predictors were not significant.

Women teachers were more likely to receive safety questions (*OR* = 3.26, *χ2*(1, N = 85) = 6.89, *p* < .01). Children in larger classes asked more questions about vaccination (*OR* = .91, *χ2*(1, N = 85) = 4.22, p < .05). Children in private schools (*OR* = .38, *χ2*(1, *N* = 85) = 5.09, *p* < .05) and higher grades (*OR* = .44, *χ2*(1, N = 85) = 5.96, *p* < .05) were less likely to ask about preventive measures. Age was not significant.

To summarize and highlight similarities and differences between Türkiye and the US, based on teacher reports on children’s questions, it was found that the form of the questions was similar across countries with one exception: children in the US asked more “why” and “how” questions than children in Türkiye. Regarding content, it was observed similarities in questions; children mostly inquired about preventive measures and the virus. Different demographic factors were associated with children’s questions in two contexts. In the Turkish sociocultural context, children’s age and the type of school they attend were important factors. Older children and children attending private schools asked more explanation-seeking questions to their teachers. Similarly, in the US, the content of children’s questions changed with their grade level, class size and type of school. Besides, in the US, teachers’ characteristics (e.g., gender) appeared to be a factor associated with children’s question form and content.

### Teachers’ responses

Teachers in Türkiye reported 356 responses, while teachers in the US reported 272 responses. As some codes were not mutually exclusive, the number of coded responses was greater (386 coded responses in the Turkish data and 300 coded responses in the US data).

#### Teachers in Türkiye

Teachers gave realistic responses to children’s questions (*n* = 232, 60.1%), followed by reassurance (*n* = 42, 10.9%). About 20.4% (*n* = 79) of the responses given by the teachers did not include any elaboration (short yes/no responses).

Teachers of higher grades reported fewer realistic answers (*OR* = .42, *χ2*(1, *N* = 110) = 7.61, *p* < .01) and were more likely to report no elaboration (*OR* = 4.63, *χ2*(1, *N* = 110) = 12.35, *p* < .001). Teachers in private schools reported more realistic answers (*OR* = 4.16, *χ2* (1, *N* = 110) = 4.18, *p* < .05). Women teachers were more likely to provide reassurance (*OR* = 3.72, *χ2* (1, N = 110) = 5.20, *p* < .05). Other predictors were not significant.

Teachers provided 201 causal explanations (49.1% of responses). These explanations referenced prior causes (*n* = 93, 46.3%), conditionals (*n* = 44, 21.9%), mechanisms (*n* = 26, 12.9%), consequences (*n* = 34, 16.9%), and precondition (*n* = 4, 2%).

Teachers with COVID-19-related school training were less likely to mention prior causes (*OR* = .43, *χ2* (1, *N* = 91) = 5.20, *p* < .05) and more likely to mention consequences (*OR* = 6.98, *χ2* (1, *N* = 91) = 5.80, *p* < .05). Teachers of higher grades were more likely to mention consequences (*OR* = .37, *χ2* (1, *N* = 91) = 7.20, *p* < .01). Other predictors were not significant.

#### Teachers in the US

Teachers provided realistic answers (*n* = 112, 35.9%), followed by reassurance (*n* = 26, 8.3%), authority (*n* = 13, 4.2%), religion (*n* = 1, 0.3%), and supernatural (*n* = 1, 0.3%). Almost half of the teachers provided no elaboration (*n* = 138, 44.2%).

Younger teachers were more likely to make no elaboration (*OR* = .93, *χ2*(1, *N* = 77) = 5.25, *p* < .05) and less likely to provide reassurance (*OR* = 1.13, *χ2*(1, *N* = 77) = 5.56, *p* < .05). Teachers in private schools were more likely to give realistic responses (*OR* = 4.68, *χ2* (1, *N* = 77) = 5.13, *p* < .05). Other predictors were not significant.

Teachers provided 133 explanations; most of them were about consequences (*n* = 80, 47.6%), conditionals (*n* = 44, 26.2%), mechanisms (*n* = 24, 14.3%), prior causes (*n* = 18, 10.7%), and preconditions (*n* = 1.2%). Teachers who felt having enough knowledge about the pandemic were more likely to mention prior causes (*OR* = 4.40, *χ2*(1, *N* = 77) = 4.92, *p* < .05). Teachers of higher grades (*OR* = .16, *χ2*(1, *N* = 77) = 4.61, *p* < .05) and who attended COVID-19-related school training (*OR* = .10, *χ2*(1, *N* = 77) = 4.94, *p* < .05) were less likely to mention prior causes. Teachers in private schools were more likely to mention mechanisms (*OR* = 4.15, *χ2*(1, *N* = 77) = 5.72, *p* < .05). Other predictors were not significant.

Our analysis of teachers’ responses revealed some key findings. In both contexts, the teacher provided realistic responses to children’s questions, and the percentage of realistic responses was higher in Türkiye than in the US. On the other hand, there were more no-elaboration responses (short yes/no responses) in the US than in Türkiye. Despite the low frequency of children’s explanation-seeking questions in Türkiye, teachers provided explanations and realistic responses. With regard to the demographic factors, participating in training and teaching higher grade levels were associated with the type of responses provided by teachers both in Türkiye and in the US. On the other hand, in the US, teachers who perceived themselves as having enough knowledge were more likely to mention prior causes than other teachers. Besides, teachers in private schools were more likely to provide mechanistic explanations to children’s questions.

### Relation between children’s questions and teachers’ answers

#### Turkish teachers

It was examined whether teachers’ responses differed by children’s question types. Lifestyle change questions (n = 14) were excluded from this analysis as they were only a few questions in this category. GLMMs with question types as predictors showed teachers were less likely to provide consequence explanations to children’s yes/no (*OR* = .09, *χ2* (1, N = 97) = 7.74, *p* < .01) and what questions (*OR* = .09, *χ2* (1, *N* = 97) = 7.29, *p* < .01). Teachers were less likely to give conditional explanations to children’s how/why questions (*OR* = .11, *χ2* (1, *N* = 97) = 6.16, *p* < .05). When whether the content of teacher’s responses differed by the content of children’s questions was examined, it was found that teachers were more likely to give realistic answers to questions about preventive measures (*OR* = 5.07, *χ2* (1, *N* = 119) = 4.4, *p* < .05). The teachers tended to provide reassurance for children’s questions about safety (*OR* = 13.62, *χ2* (1, *N* = 119) = 5.09, *p* < .05). Teachers were more likely to make no elaboration when children asked about vaccination (*OR* = 11.16, *χ2* (1, *N* = 119) = 4.78, *p* < .05).

Teachers were more likely to provide explanations to children’s explanation-seeking questions (*OR* = 4.49, *χ2*(1, *N* = 97) = 8.73, *p* < .01). Teachers were more likely to give mechanistic (*OR* = 11.16, *χ2* (1, *N* = 97) = 9.97, *p* < .01) and consequence explanations (*OR* = 6.77, *χ2* (1, *N* = 97) = 9.56, *p* < .01) if children asked how/why than yes/no questions.

#### US teachers

It was found that teachers were more likely to give realistic answers when children asked why questions (*OR* = 2.53, *χ2* (1, *N* = 92) = 9.30, *p* < .01) and less likely to give realistic answers when children asked what questions (*OR* = .43, *χ2* (1, *N* = 92) = 4.64, *p* < .05). Teachers were less likely to make no elaboration to children’s questions about preventive measures (*OR* = .19, *χ2*(1, *N* = 92) = 4.75, *p* < .05) when it was examined whether the teachers’ answer contents differed by children’s question contents.

Finally, teachers were more likely to provide explanations to how/why questions (*OR* = 1.69, χ2(1, N = 92) = 7.27, p < .01), and teachers were more likely to give consequence explanations to why questions (*OR* = 3.10, *χ2* (1, *N* = 92) = 3.75, *p* = .05).

Overall, both in Türkiye and in the US, teachers were more likely to provide explanations to children’s explanation-seeking “why” and “how” questions. Teachers were more likely to provide complete and realistic responses to questions about preventive measures. In Türkiye, teachers were more likely to provide reassurance when children asked questions about safety. However, they avoided providing elaborations when children asked questions about vaccination.

## Discussion

This study examined the form and content of children’s questions about the COVID-19 pandemic and the quality of responses teachers provided to these questions in Türkiye and the US. In addition, it investigated the role of demographic factors in age, gender, and grade level in children’s question-asking behavior and teachers’ responses. Conducting this investigation in Türkiye and in the US provided insight into similarities and differences in how children seek information about the pandemic and how teachers inform children when responding to their questions. Below, the findings are discussed based on our research questions and hypotheses.

### Children’s questions

Confirming our hypotheses, the findings showed that the content of children’s questions about COVID-19 was similar in Türkiye and the US; they asked about preventive measures, time, and safety. However, when it comes to the form of questions, children in Türkiye asked yes/no and what questions frequently, but explanation-seeking questions were not very common. Children in the US asked yes/no and what questions at similar levels, but explanation-seeking questions occurred in one-third of their questions to teachers. This finding was consistent with previous findings examining children’s information-seeking question-asking behavior in the US and in Türkiye [3, 5, 22].

Contrary to our expectations regarding grade level differences, we found that in Türkiye, younger children asked more explanation-seeking questions than older children, while older children asked yes/no questions. Additionally, although we did not have any specific hypotheses about school type, in the Turkish sample, children attending private schools asked more explanation-seeking questions than children attending public schools. We did not observe these relations in the US sample, which might be due to the higher rate of explanation-seeking questions. These findings suggest that the difference in explanation-seeking questions between children in Türkiye and the US is smaller for younger children. However, with experience and socialization, children in Türkiye may tend to ask fewer explanation-seeking questions at higher grades. Our hypothesis that the most common content of children’s questions would be virus and precaution-related questions was supported in both Türkiye and in the US, aligning with studies examining children’s questions about the pandemic with parents [1]. However, we did not observe any grade-level differences. Overall, the findings corroborate earlier research indicating sociocultural influences on children’s questions [5, 21]. Moreover, these findings also suggest that while children’s concerns about the virus were similar (i.e., trying to understand the virus and preventive measures), how they formed their questions and inquired about this content showed differences.

Moreover, the findings also showed that in Türkiye, teachers in public schools report fewer explanation-seeking questions than teachers in private schools, aligning with previous findings showing SES differences. Given that attending a private school requires paying tuition, we can consider enrollment in private schools as an indicator of higher SES. Thus, this finding replicates previous work on parent-child conversations, showing that children from high-SES families ask more explanation-seeking questions than children from low-SES families [6, 22, 37]. However, the mechanism behind this difference remains an open question. One possibility is that children from middle-SES receive more informative answers to their questions at home and school and have more opportunities to engage in extended dialogues with adults, promoting their exploratory behavior [6, 37]. Another possibility is that differences in parent and teacher mindsets in middle-SES and low-SES backgrounds influence their socialization practices [20, 38]. Thus, while we conducted this study in two countries, we have glossed over important differences within these contexts depending on factors such as SES (whether a school is private), age, and training. Future research should examine differences within and across cultures.

### Teachers’ responses

The findings showed that teachers in both countries provided realistic responses to children’s questions. It is also worth noting that teachers provided explanations at similar rates in both countries, suggesting that children in Türkiye might realize that they do not need to actively seek out this information as teachers tend to provide it. Supporting our hypotheses, teachers in Türkiye were more likely to refer to rules and authority than teachers in the US, reflecting sociocultural norms emphasizing interdependence and hierarchy [19, 20]. Moreover, confirming our expectations, we found that teachers who perceived themselves as more knowledgeable were more likely to provide realistic responses [1, 3]. We also found that demographic factors such as teacher’s age and gender influenced children’s questions in the US and in Türkiye. For instance, women teachers received more safety-related questions. In addition, women teachers in Türkiye provided more reassurance in their responses than men teachers. These findings suggest that the type and content of questions and responses differ based on context and the informant’s characteristics. We only examined several demographic factors in this study but future work should examine how specific informant characteristics children’s information-seeking behaviors.

In both countries, teachers in private schools were more likely to provide realistic responses than teachers in public schools. This finding matches with findings from children’s questions that children attending private schools ask more explanation-seeking questions. When we examined the causal content of teachers’ responses, we found that teachers’ knowledge about the coronavirus (in the US) and COVID-19-related training (in Türkiye) were related to responses with prior causes. Consequently, not only teacher characteristics but also child characteristics influenced teacher responses.

Observing that Turkish teachers provided realistic responses and causal explanations as frequently as teachers from the US showed that even if children in Türkiye do not ask as many explanation-seeking questions as children in the US, they still hear similar, if not more, causal explanations as their US counterparts. As mentioned, children might be sensitive to these patterns and realize that they do not need to ask why/how questions as teachers provide explanations regardless. This finding corroborates early work on parent-child conversations, showing that parents provide explanations in the absence of questions [39]. Future work should explore how children shift their questions depending on whether the informant voluntarily provides explanations even when not asked.

Verifying our hypotheses regarding the relation between children’s questions and teachers’ responses, we found that teachers were more likely to provide explanations when children’s questions were explanation-seeking. The contingency between questions and answers aligned with previous findings [4]. Moreover, teachers in Türkiye were more likely to provide reassurance to questions about safety, showing that they were not only trying to give factual information but also attempting to comfort the child about potentially stressful topics. This shows that teachers have multiple goals when answering children’s questions.

### Limitations

This study’s limitation includes its reliance on teachers’ retrospective self-reports. This could mean that teachers did not report all the questions from children but rather questions easy to remember due to frequency, recency, or saliency. Moreover, teachers’ responses could include optimal responses rather than actual responses. Thus, naturalistic observation is necessary to understand how children and teachers discuss these topics in the classroom. However, as COVID-19-related questions are likely relatively infrequent and the pandemic was unfolding in real-time, collecting observational classroom data may not be all that feasible.

### Conclusion

In conclusion, this study advances our understanding of the development of question-asking about viruses and biological reasoning within the context of COVID-19 but with broader implications for children’s learning about health and biology-related topics. These findings also highlight the importance of teacher knowledge and teacher training in providing realistic responses to children’s questions at the time of a global health crisis like COVID-19 pandemic. Thus, in the future, it is important to provide teachers with professional development programs to equip them with the necessary knowledge and resources to respond informatively to children’s questions. Moreover, considering the similarities and differences between the two sociocultural contexts, it is important to consider the sociocultural context when preparing teacher training programs and learning resources for children.
